# Of mice and men and larvae: *Galleria mellonella* to model the early host-pathogen interactions after fungal infection

**DOI:** 10.1080/21505594.2017.1382799

**Published:** 2017-11-10

**Authors:** Andrew M. Borman

**Affiliations:** UK National Mycology Reference Laboratory (MRL), Public Health England South-West, Bristol, UK

**Keywords:** *Candida albicans*, *Galleria mellonella*, fungal pathogenesis, host-pathogen interactions, model organism, proteomics, immunity

*Candida* species are the most common fungal causes of deep-seated and disseminated infections in immunocompromised human hosts, and are associated with high morbidity and mortality in this patient population.^1,2^ Although the incidence of invasive fungal infections caused by unusual *Candida* spp. continues to rise, to date *Candida albicans* remains the most frequently isolated *Candida* species in the clinical setting, is the principal agent of nosocomial yeast infections,^1,3^ and is widely accepted as being one of the most virulent *Candida* species.^4^ A large number of virulence and fitness factors have been identified in *C. albicans*, and include the ability to undergo yeast-hyphal transition, the expression of adhesins, invasins and hydrolytic enzymes which promote biofilm formation and tissue invasion, and rapid adaptations to changing extracellular environments (reviewed in^5^). However, since disease severity and outcome depend upon the complex interplay between the virulence of the individual fungal pathogen and the immune response of the host,^6^ study of both aspects is central to furthering our understanding of fungal pathogenesis.

A large number of animal models have been developed to allow the study of both superficial and disseminated candidosis, and murine models continue to be promoted as the gold standard for evaluating *Candida* pathogenicity.^7^ Study of the host response and the determinants of fungal virulence have been aided by such animal models since they replicate human disease with high fidelity. In addition, the availability of genetically modified or immune-depleted hosts has allowed the dissection of the principal host immune components.^8^ However, animal experimentation is constrained by major bioethical, economic and logistical issues, and the “3Rs” policy adopted by many international and governmental funding agencies encourages the development of alternative model systems that do not have associated bioethical issues. Amongst alternative model systems for studying microbial pathogenesis, two insects in particular have been extensively used over recent years: the fruit fly *Drosophila melanogaster* and the larvae of the greater wax moth *Galleria mellonella* (reviewed in^9^). Advantages include inexpensive and easy breeding in large numbers, relatively simple maintenance in the laboratory, and ease of inoculation. In addition, insect innate immune systems at the cellular and humoral level are structurally and functionally very similar to the immune system in mammals, allowing results obtained in insect models to be easily translated to humans.^10,11^ In insects, pathogens are recognised by pathogen recognition receptors,^12,13^ phagocytosed by hemocytes which are functional equivalents of mammalian neutrophils,^14^ and eliminated using reactive oxygen species as in mammals.^15^ Insects also synthesise a range of antimicrobial peptides, many of which are evolutionarily conserved between invertebrates and mammals.^16,17^

The ability to infect *Drosophila* with fungi, bacteria and viruses,^18^ the availability of a completed genome sequence^19^ and extensive gene microarrays^20^ and the ease of genetic manipulation have made this organism a model system for infection studies.^21^ Indeed, research with *Drosophila* has elucidated many of the central mechanisms of anti-pathogen immunity,^21,22^ including the discovery that the Toll signalling pathway was central to an effective antifungal host response against *Aspergillus fumigatus*.^23^ However, despite the lack of equivalent genomic resources, *Galleria* also has specific advantages for the study of human pathogens and is an increasingly popular choice for the investigation of the determinants of fungal pathogenesis. Wild-type larvae are susceptible to human-pathogenic fungi without the need for manipulation of the Toll pathway and genetic crossing, and can be housed in simple petri dishes in the laboratory. The larger size of the larvae as compared to *Drosophila* potentially allows multiple inoculations into the same organism, the use of more accurately quantified inocula with less specialised equipment, and more sophisticated and varied end-point analyses (melanisation, fungal burden, larval death, alterations in hemocyte composition and larval genomic/proteomic changes).^24,25^ Moreover, *Galleria* larvae can be maintained at human physiological temperatures and above, an important consideration when temperature-dependent virulence factors are involved.^26,27^ In addition, several studies have demonstrated that pre-exposure of *G. mellonella* to sub-lethal doses of fungi protected the larvae from subsequent lethal challenges, in part by inducing the production of protective antimicrobial peptides, indicating that *Galleria* is able to assess the extent of fungal infection and differentially activate cellular and humoral responses.^28,29^ To date, *Galleria mellonella* has been successfully employed in studies comparing virulence of different fungi,^30,31^ elucidation of virulence factors,^32-34^ antifungal drug response and resistance,^35-37^ combination therapy^38^ and pharmacokinetics,^39^ alternative antifungal therapies^40,41^ and probiotics.^42,43^ In addition, this invertebrate model is apparently capable of reproducing clinical features seen with human infection with remarkable fidelity, as evidenced by the demonstration of fungal grain development in larvae infected with an agent of eumycetoma^44^ and aggregates of immobilised *Listeria* bacteria on the brains of larvae that are similar to those seen on the brains of infected humans.^45^

In a recent issue of *Virulence*,^46^ the group of Kavanagh, one of the leading proponents of the use of *Galleria* as a fungal infection model has further strengthened the argument, by exploiting the recently published transcriptome and immune-gene repertoire of *Galleria*^47^ to examine the early cellular and humoral responses of larvae after infection by *Candida albicans*. The authors infected larvae with a dose of *C. albicans* that permitted larval survival for 24 hours and followed fungal burden, changes in hemocyte numbers and population structure and employed semi-quantitative shotgun proteomics to analyse the larval response early (6 hours) and late (24 hours) after infection. Their analyses revealed a biphasic response to infection. In the early acute phase, hemocyte density, antimicrobial peptides and immune proteins all increased significantly in abundance with a concomitant reduction in larval fungal burden. The late phase (6-24 hours) conversely was marked by extensive fungal proliferation, reduction of the overall hemocyte population with an increase in the number of granular hemocytes and proteomic changes indicative of cellular stress, tissue damage, chemo-protection and sequestration of key immune related proteins in immune-fungal complexes. These data are highly suggestive of a sophisticated and orchestrated response in which early stages are designed to gauge the extent of fungal infection via non-specific immune reactions (antimicrobial peptides, melanisation) and determine the full immune response required, followed by the later unleashing of a large specific response involving increased production of antimicrobial peptides and phagocytic cells. What makes the study more important is that the initial cellular and humoral responses observed in *Galleria* appear very similar to those that are seen in murine models of invasive candidiasis, where an initial massive innate immune response involving cellular recruitment, complement activation and opsonisation occurs early in the neutrophil-poor kidneys of infected animals prior to wide scale peripheral neutrophilia.^48^ Moreover, this latest addition to the *Galleria* literature clearly demonstrates that the lack of whole genome data for this model insect does not prevent a detailed exploration of host-pathogen interactions.
